# Infant-Type Hemispheric Glioma With NTRK Fusion Responds to Larotrectinib

**DOI:** 10.7759/cureus.114069

**Published:** 2026-08-06

**Authors:** Pragya Virendrakumar Jain, Sarah Rumler, Paula North

**Affiliations:** 1 Pathology, Medical College of Wisconsin, Milwaukee, USA; 2 Pediatric Neuro-Oncology Program, Division of Hematology, Oncology, and Blood and Marrow Transplant, Medical College of Wisconsin, Milwaukee, USA

**Keywords:** alk, infant-type high grade glioma, larotrectinib, met1, ntrk, pediatric type diffuse glioma, ros1

## Abstract

Infant-type hemispheric glioma is a distinct form of high-grade glioma occurring in infancy and early childhood. It arises in the cerebral hemispheres and is characterized by recurrent molecular alterations involving genes such as NTRK, ROS1, MET, and ALK. We present the case of a four-month-old infant with a large solid-cystic parietal-occipital hemispheric mass. Histologic examination revealed a cellular, poorly differentiated tumor with areas of necrosis and prominent desmoplasia. DNA methylation profiling supported classification as infant-type hemispheric glioma, and next-generation sequencing identified a TPM3::NTRK1 fusion. An integrated diagnosis of infant-type hemispheric glioma was made. Following surgical resection, targeted therapy with an NTRK inhibitor was initiated, resulting in a sustained clinical and radiologic response. This case underscores the importance of integrated histologic and molecular evaluation in establishing the diagnosis and guiding management of these tumors, as well as the clinical relevance of identifying targetable alterations.

## Introduction

Central nervous system (CNS) tumors in children are rare and varied in type, with approximately 10% of these presenting in the first six months of life [1]. Many of these infantile brain tumors are gliomas. These gliomas typically present in infancy with variable clinical features and are usually large tumors [2-8]. Historically, diagnosis of pediatric CNS tumors has heavily relied on histological features, incorporating few supportive immunohistochemical and molecular findings. However, the 2021 WHO classification of central nervous system (CNS) tumors has codified an important new dimension for diagnosis and management of these tumors. With better molecular subclassification and the availability of targeted therapeutic drugs, the concept of integrated diagnosis and a multidisciplinary approach for patient management is gaining popularity [2]. We hereby present the case of an infant presenting with a cerebral infant-type hemispheric glioma that, based on its undifferentiated histology alone, would have been classified among clinically higher-grade pediatric diffuse high-grade gliomas (HGG). However, under the latest WHO classification, this tumor could be further subtyped through molecular discovery, empowering targeted therapy and revealing improved prognosis.

## Case presentation

A four-month-old female child with a history of prematurity presented with three days of vomiting and a rapidly increasing head size. On physical exam, her head circumference was massively increased. Parents reported a history of some feeding difficulty. They denied any other significant medical or surgical history.

**Figure 1:**
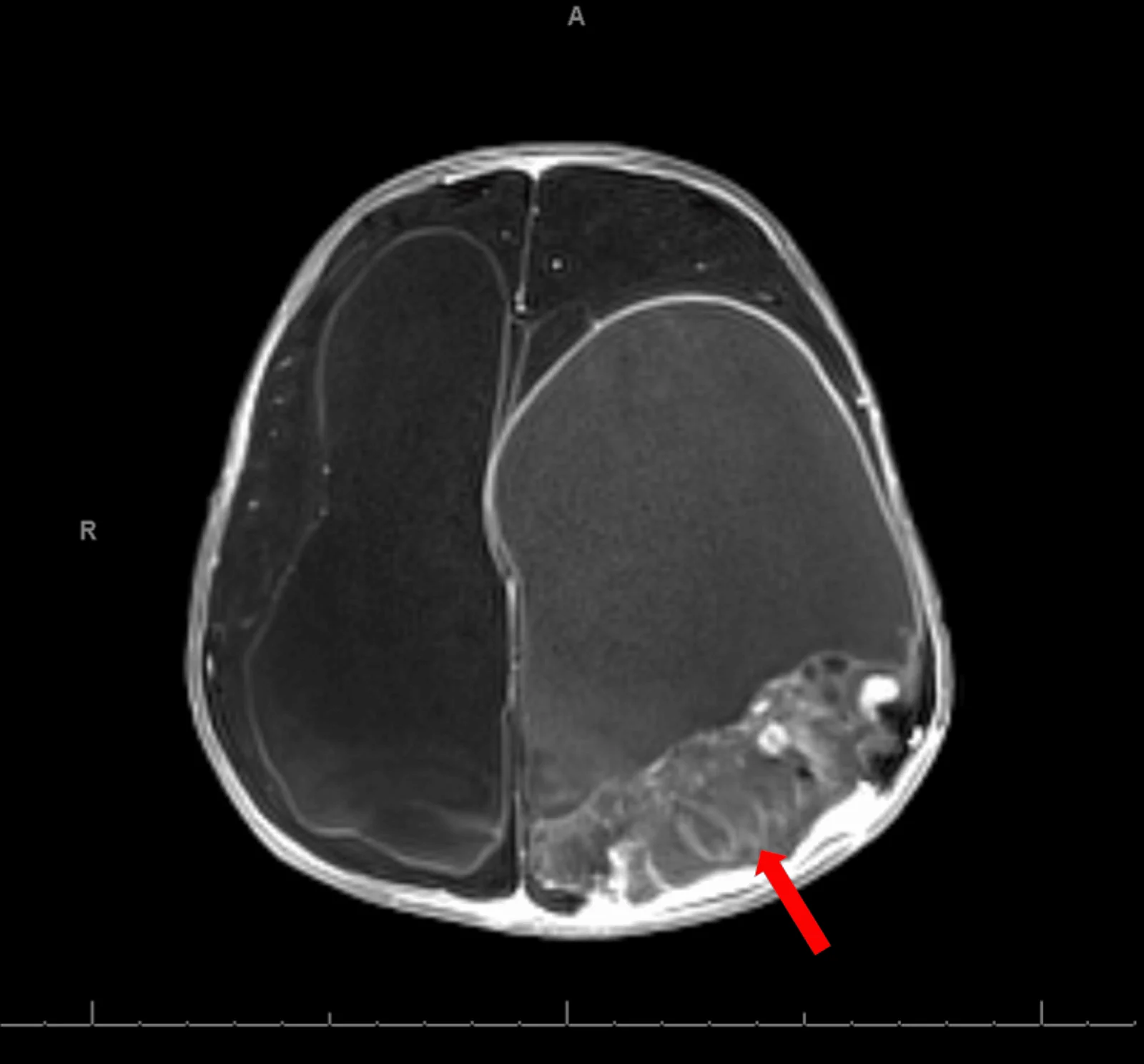
Pre-operative MRI (T1 post-contrast). Complex posterior parietal-occipital mass measuring 9.3 x 3.2 x 9.5 cm (indicated by red arrow). There is heterogeneous contrast enhancement, significant intratumoral vascularity, cystic areas, increased blood perfusion, and diffusion restriction (suggestive of a high-grade lesion). The mass causes marked right-to-left midline shift, resulting in significant mass effect on the brainstem and ventricles.

On MRI, an 8.1 x 12.1 x 10.8 cm complex, solid-cystic left parietal-occipital hemispheric mass lesion causing a 13 mm right-to-left midline shift was present. There was marked mass effect upon the brainstem and ventricles causing hydrocephalus (Figure 1).

**Figure 2:**
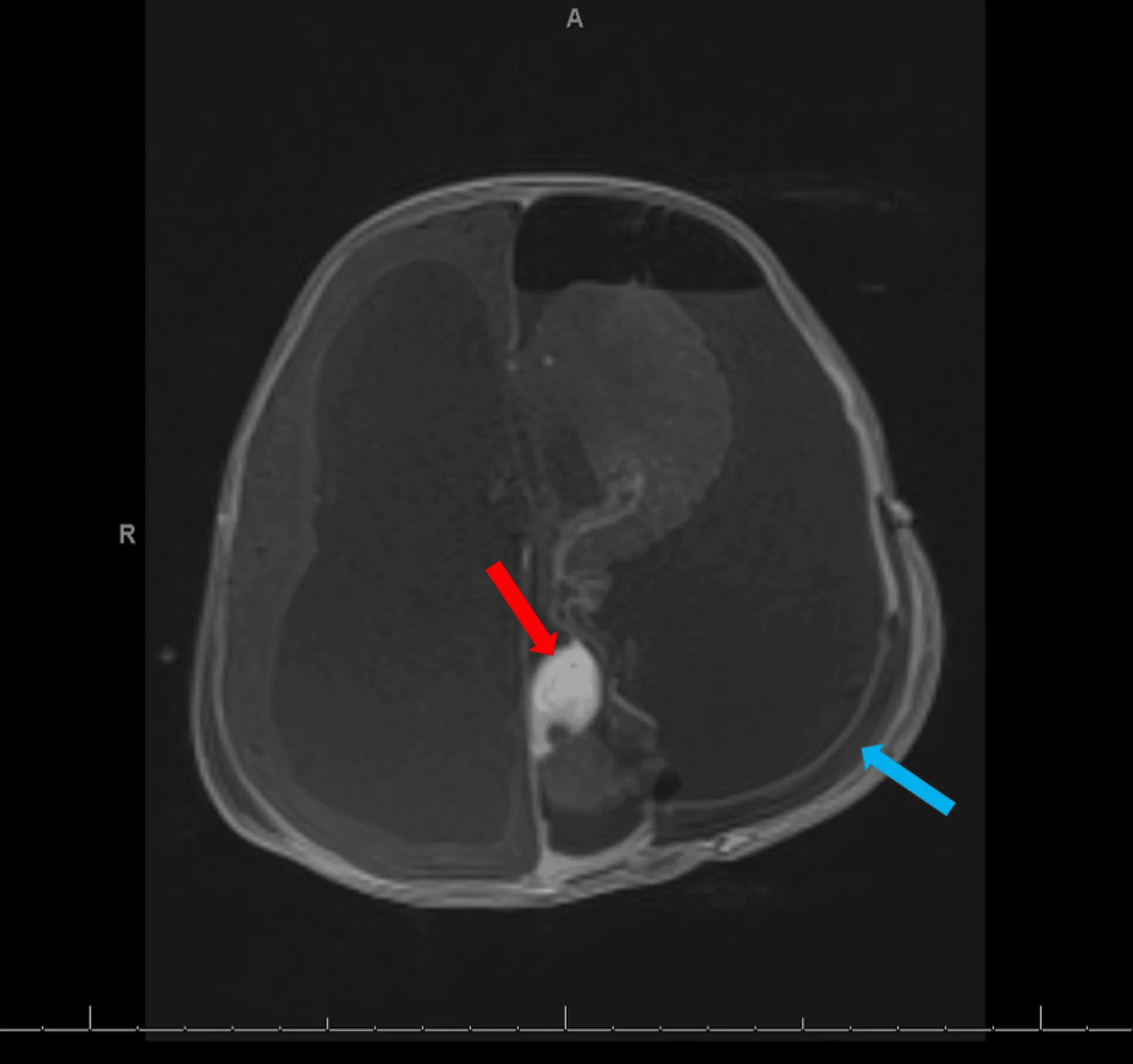
Immediately post-operative MRI (T1 post-contrast). Post-operative change with hematoma around the resection bed (red arrow) and an extradural fluid collection (blue arrow). No evidence of residual lesion identified.

On imaging, the differential diagnosis included embryonal tumors, such as atypical teratoid/rhabdoid tumor (ATRT), embryonal tumor with multilayered rosettes (ETMR), astroblastoma, or teratoma; however, other etiologies could not be excluded. Craniotomy was performed for tumor resection (Figure 2); a small fragment of tissue was submitted for intra-operative frozen section, and the remaining tissue was submitted for histological evaluation and molecular studies.

**Figure 3:**
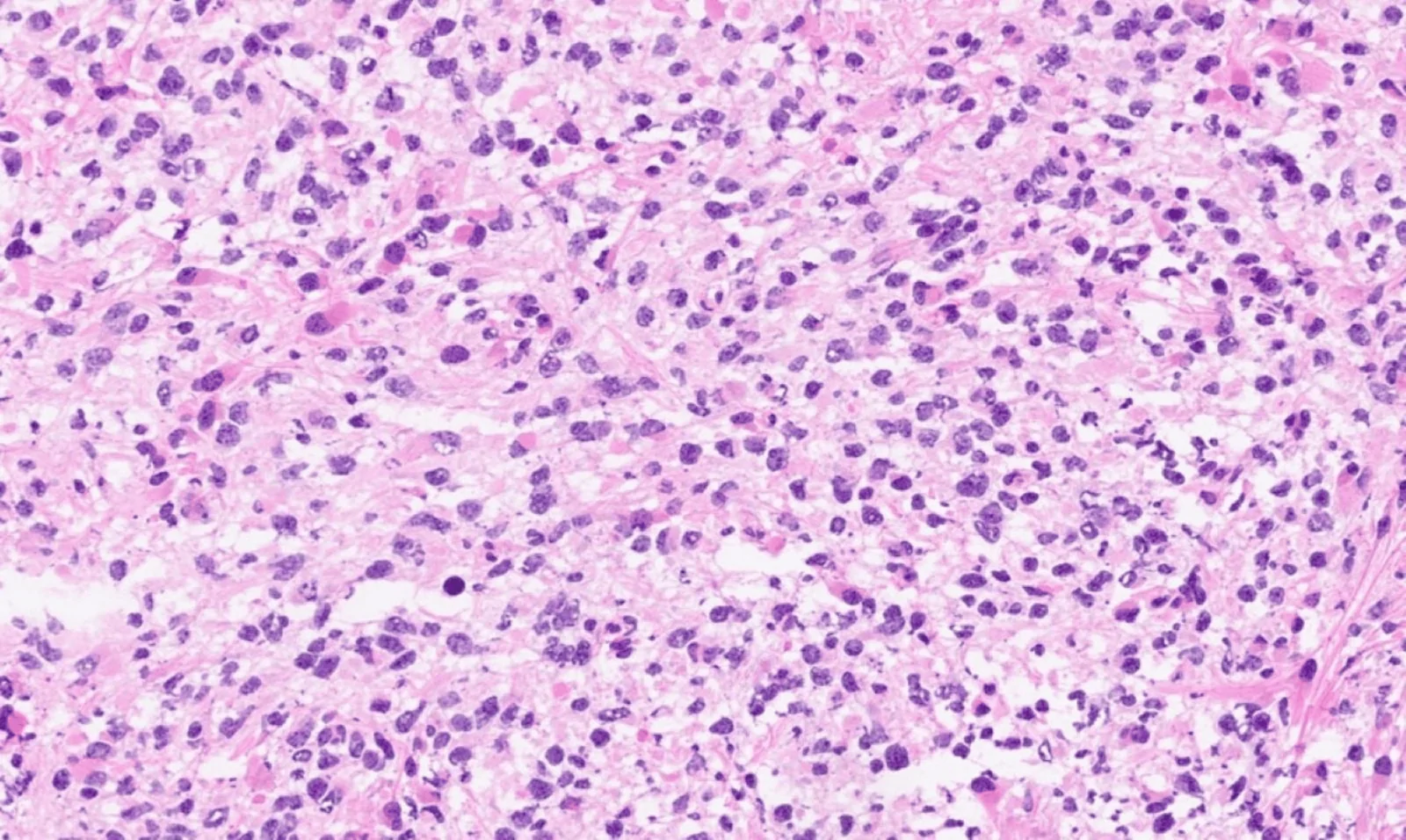
Microscopic image of infant-type hemispheric glioma: frozen section, H&E stain, 20x. Poorly differentiated cells.

**Figure 4:**
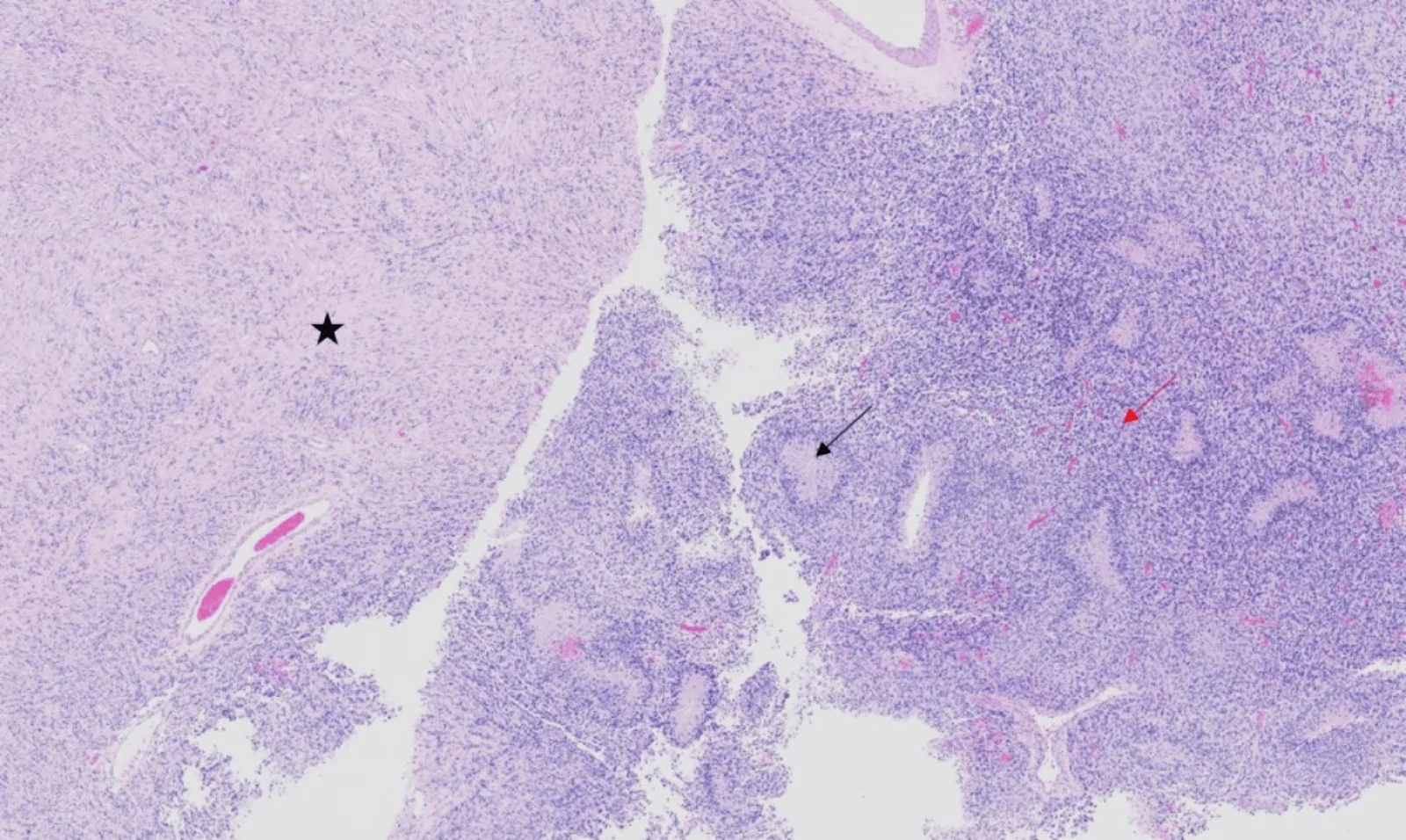
Microscopic image of infant-type hemispheric glioma, H&E stain, 4x. Poorly differentiated areas (red arrow), necrosis (black arrow), and desmoplastic stroma (black star).

**Figure 5:**
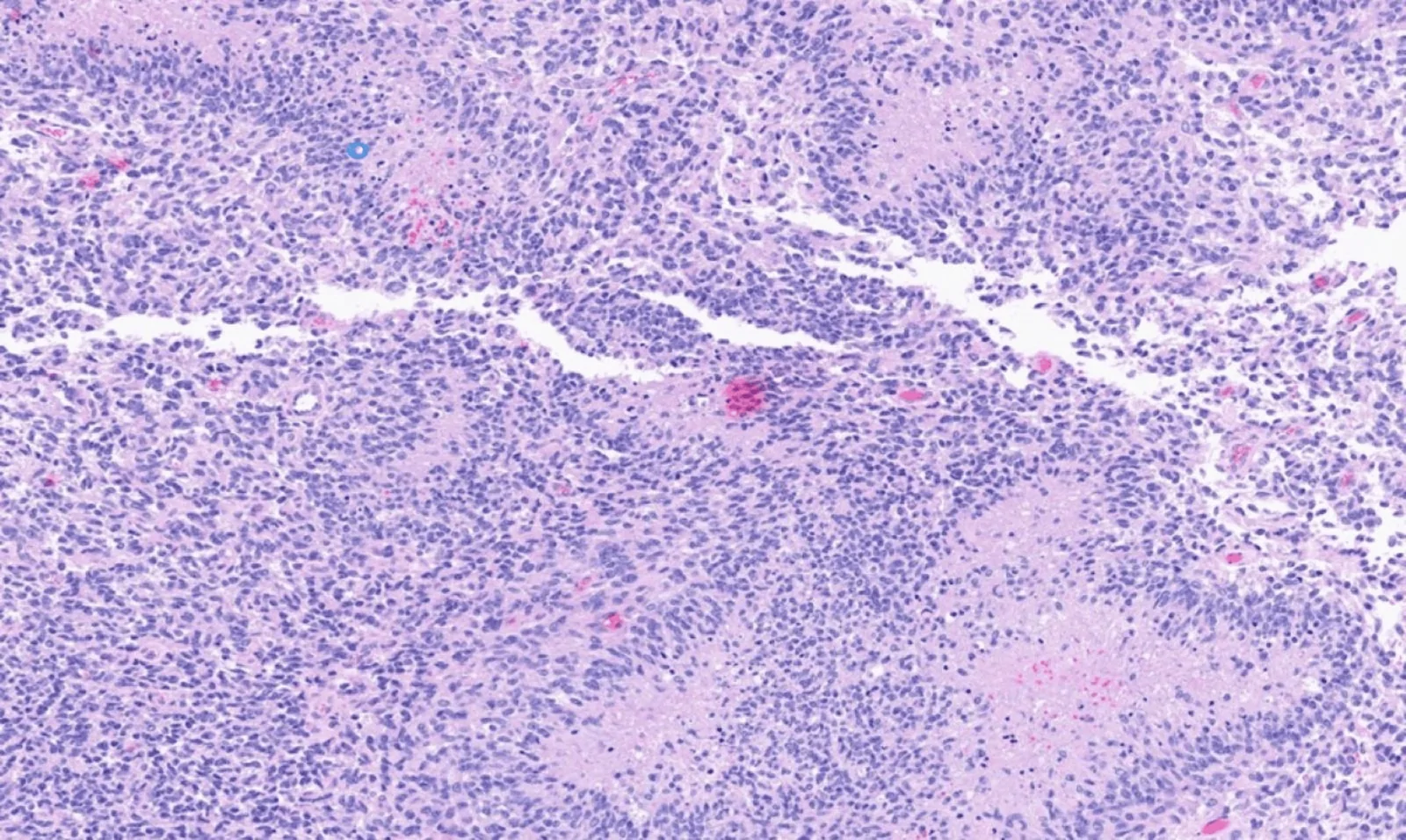
Microscopic image of infant-type hemispheric glioma, H&E stain, 40x. Palisading necrosis and poorly differentiated cells.

**Figure 6:**
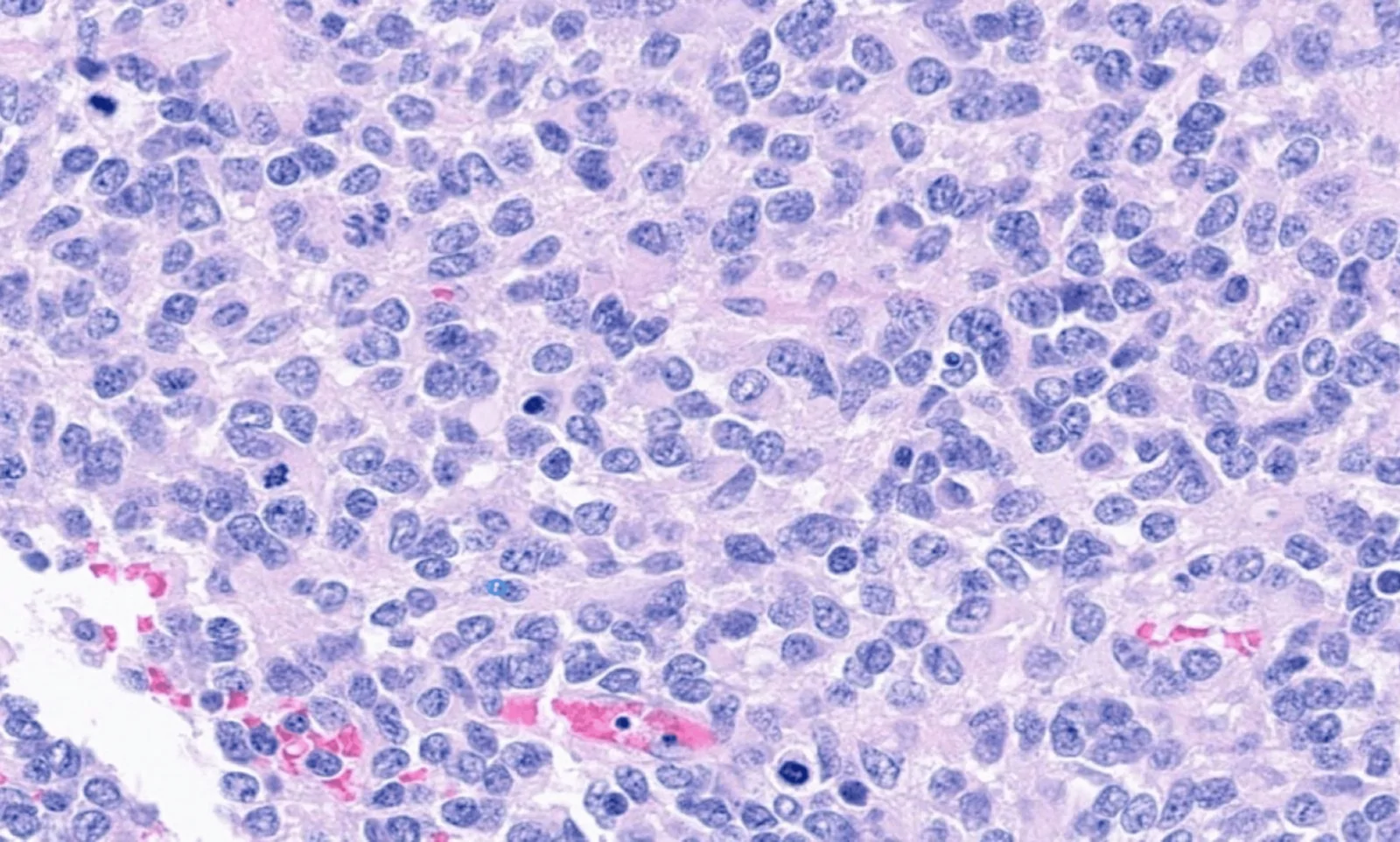
Microscopic image of infant-type hemispheric glioma, H&E stain, 40x. Poorly differentiated cells and frequent mitotic figures.

**Figure 7:**
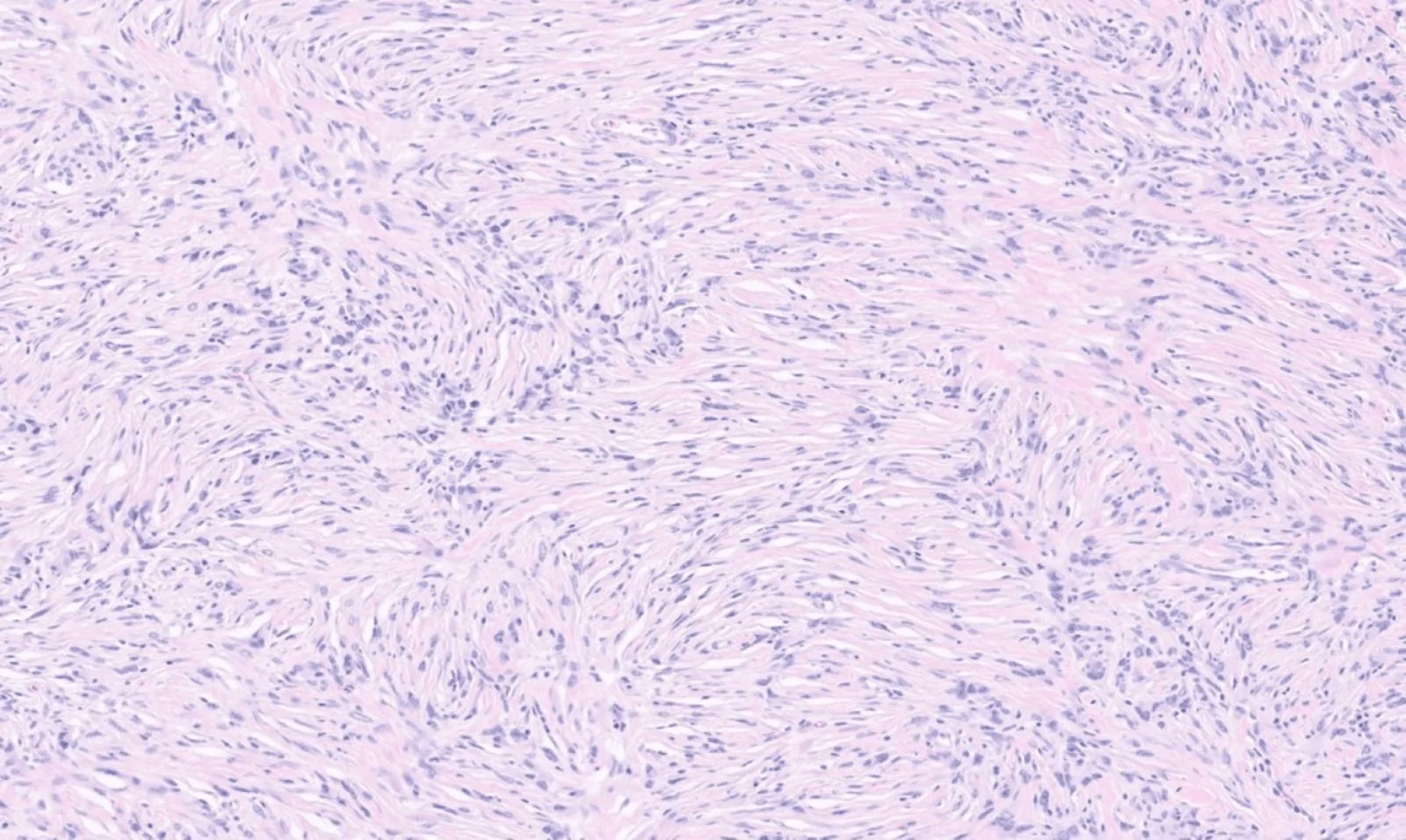
Microscopic image of infant-type hemispheric glioma, H&E stain (4x). Desmoplastic stroma with spindle-shaped cells.

Grossly, a 9.0 x 6.0 x 4.4 cm aggregate of tan-red soft tissue fragments with areas of necrosis and hemorrhage was seen. Small portions of cerebral cortex and dura were also identified. Frozen and permanent sections (Figures 3-7) collectively showed sharply demarcated areas of highly cellular, poorly differentiated cellular proliferation and areas of striking desmoplasia. The desmoplastic components consisted of fibroblast-like spindle cells and pleomorphic neuroepithelial cells arranged in fascicular and whorled patterns displaying a prominent network of reticulin-positive fibers that encircled individual tumor cells. The poorly differentiated areas predominated and consisted of cellular sheets of small cells of embryonal or astroglial appearance with dark nuclei displaying scattered, focally numerous mitotic figures, and focally admixed with gemistocytic cells with abundant eosinophilic cytoplasm. The poorly differentiated areas multifocally contained foci of serpiginous palisading necrosis. Areas of cystic degeneration and accumulation of hemosiderin-laden macrophages were also noted.

**Figure 8:**
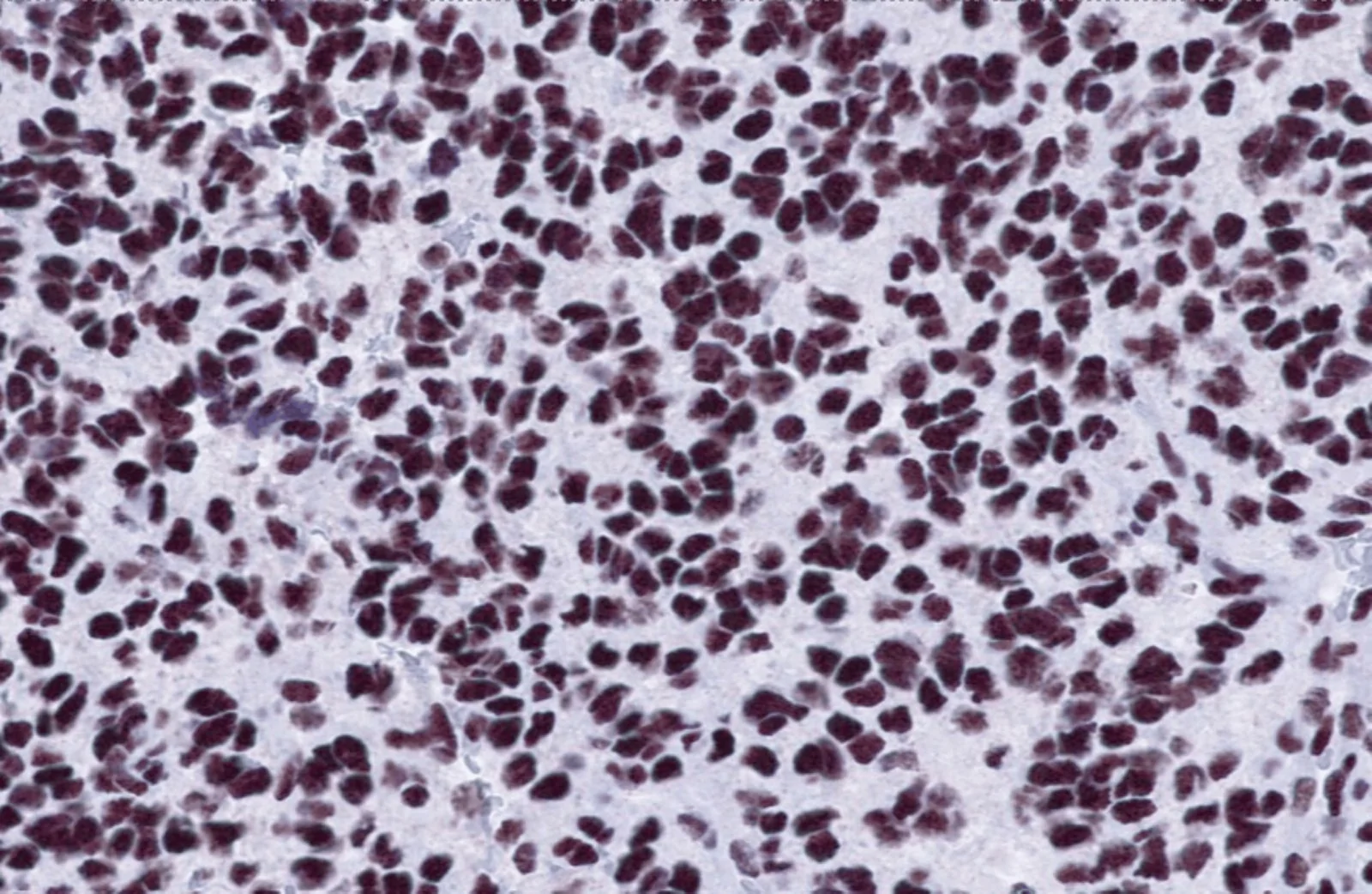
INI-1/BAF47 immunostain, 40x. Expression retained throughout the tumor.

**Figure 9:**
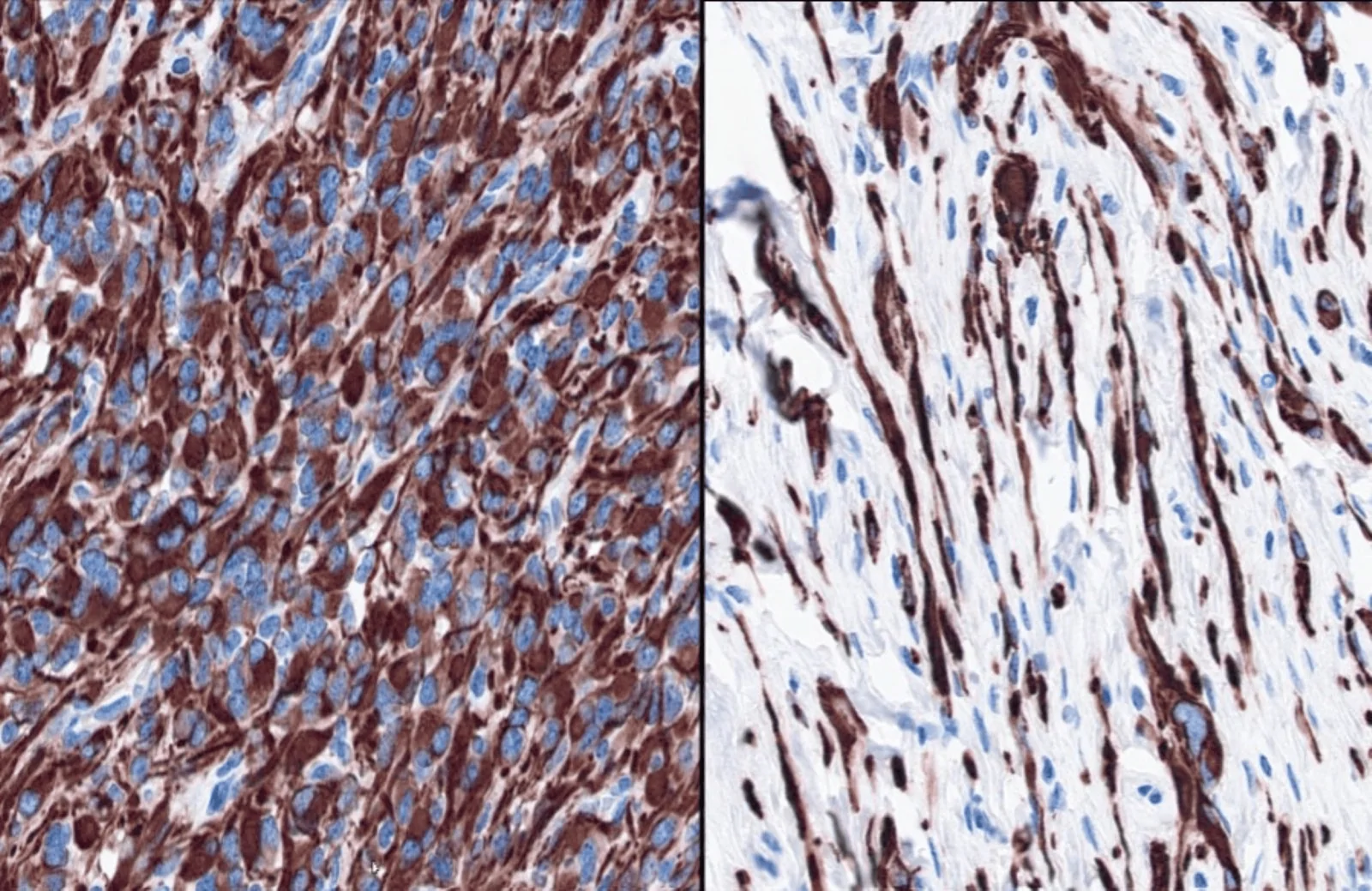
GFAP immunostain, 40x. GFAP staining strongly highlighted the poorly differentiated component (left side) and the fibroblast-like cells within the desmoplastic zones (right side). GFAP: glial fibrillary acidic protein.

**Figure 10:**
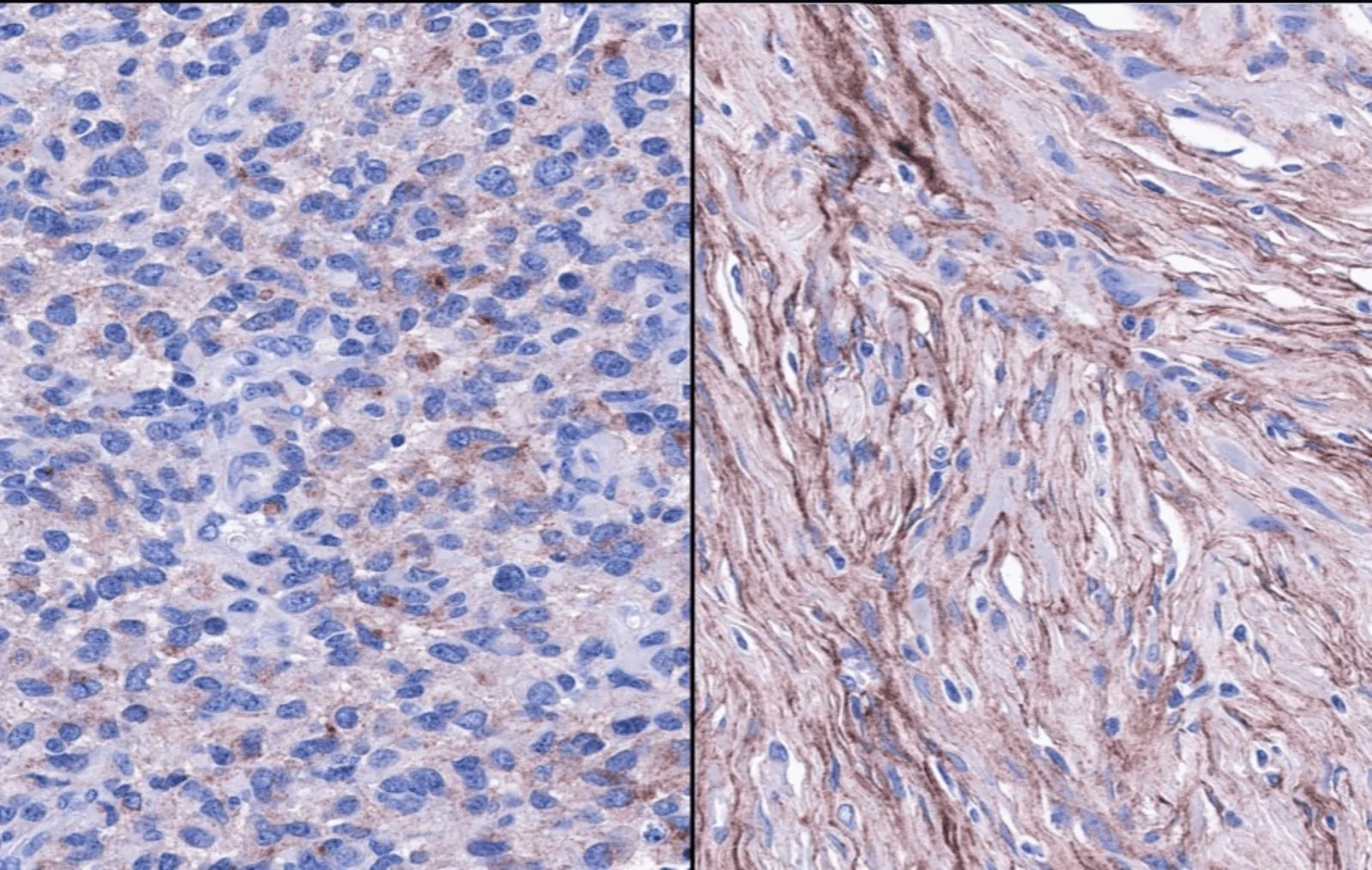
Epithelial membrane antigen (EMA) immunostain, 40x. Patchy light-to-strong positivity in the poorly differentiated component (left side) as well as the fibroblast-like cells within the desmoplastic zones (right side).

**Figure 11:**
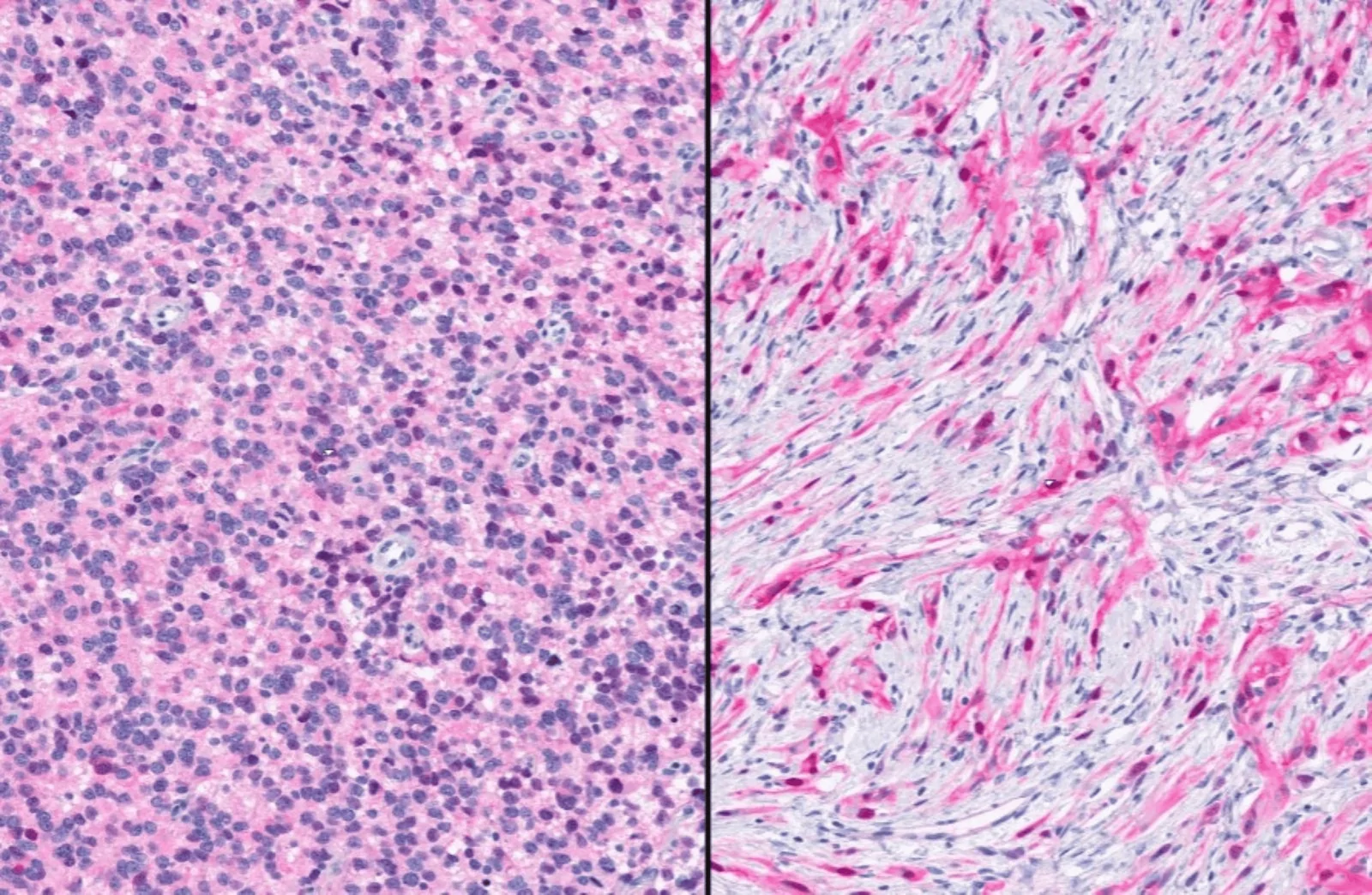
S100 immunostain, 40x. Diffuse light positivity in the poorly differentiated component (left side) and strong positivity in the fibroblast-like cells within the desmoplastic zones (right side).

**Figure 12:**
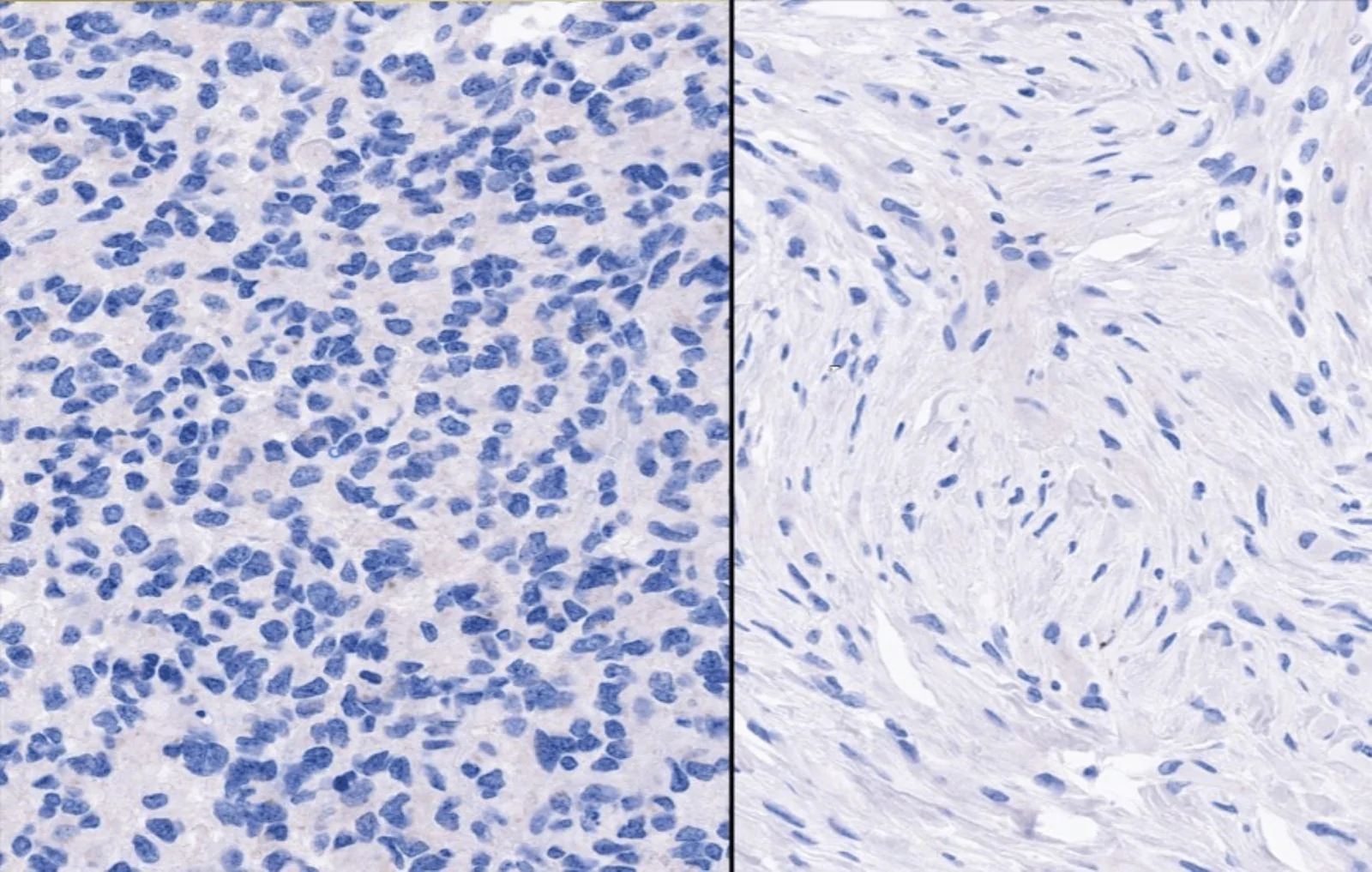
Synaptophysin immunostain, 40x. Focal weak cytoplasmic positivity (without definitive neuronal cytologic differentiation) in the poorly differentiated component and negativity in the fibroblast-like cells within the desmoplastic zones.

**Figure 13:**
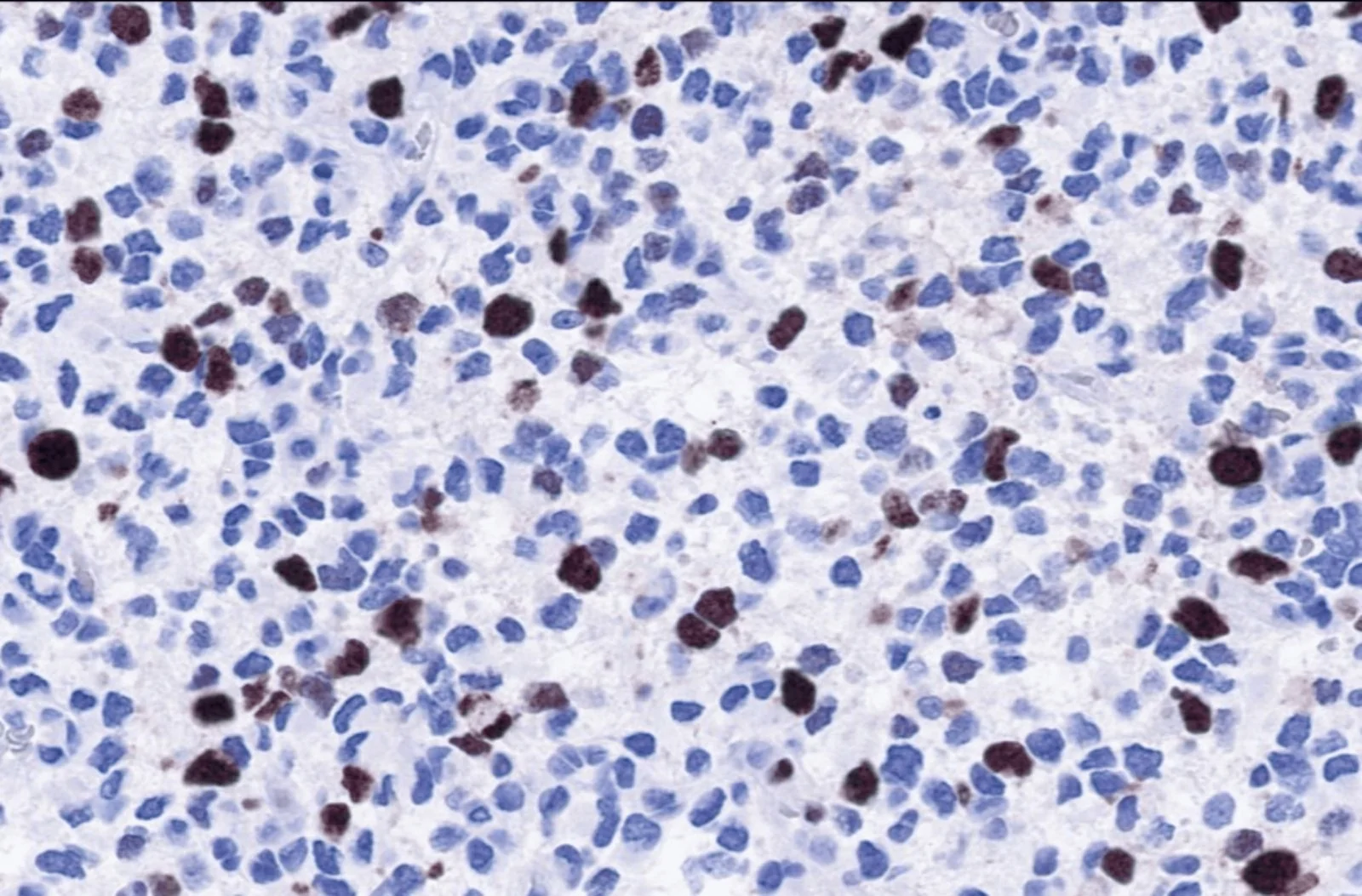
MIB-1 immunostain, 40x. Nuclear staining was essentially restricted to the poorly differentiated areas, where positivity was patchy and variable, with indices ranging from less than 0.5% to occasionally 5%.

A well-controlled gamut of immunohistochemical stains was applied. INI-1/BAF47 expression was retained throughout the tumor (Figure 8). Glial fibrillary acidic protein (GFAP) staining strongly highlighted the poorly differentiated component and the fibroblast-like cells within the desmoplastic zones (Figure 9). Epithelial membrane antigen (EMA) displayed patchy light-to-strong positivity in both the poorly differentiated component and the fibroblast-like cells within the desmoplastic zones (Figure 10). S100 showed diffuse light positivity in the poorly differentiated component and strong positivity in the fibroblast-like cells within the desmoplastic zones (Figure 11). Synaptophysin immunostain showed focal weak cytoplasmic positivity(without definitive neuronal cytologic differentiation) in the poorly differentiated component and negativity in the fibroblast-like cells within the desmoplastic zones (Figure 12). MIB-1 nuclear staining was essentially restricted to the poorly differentiated areas, where positivity was patchy and variable, with indices ranging from less than 0.5% to occasionally 5% (Figure 13).

On cytogenetic analysis, 11/55 cells were found to be tetraploid with no clonal numerical or structural abnormalities. DNA methylation profiling supported classification as infant-type hemispheric glioma with a high confidence score [8]. Next-generation sequencing showed a TPM3(7)-NTRK1(2) gene fusion, which is a common driver in infantile gliomas [8]. An integrated diagnosis of infant-type hemispheric glioma was rendered.

**Figure 14:**
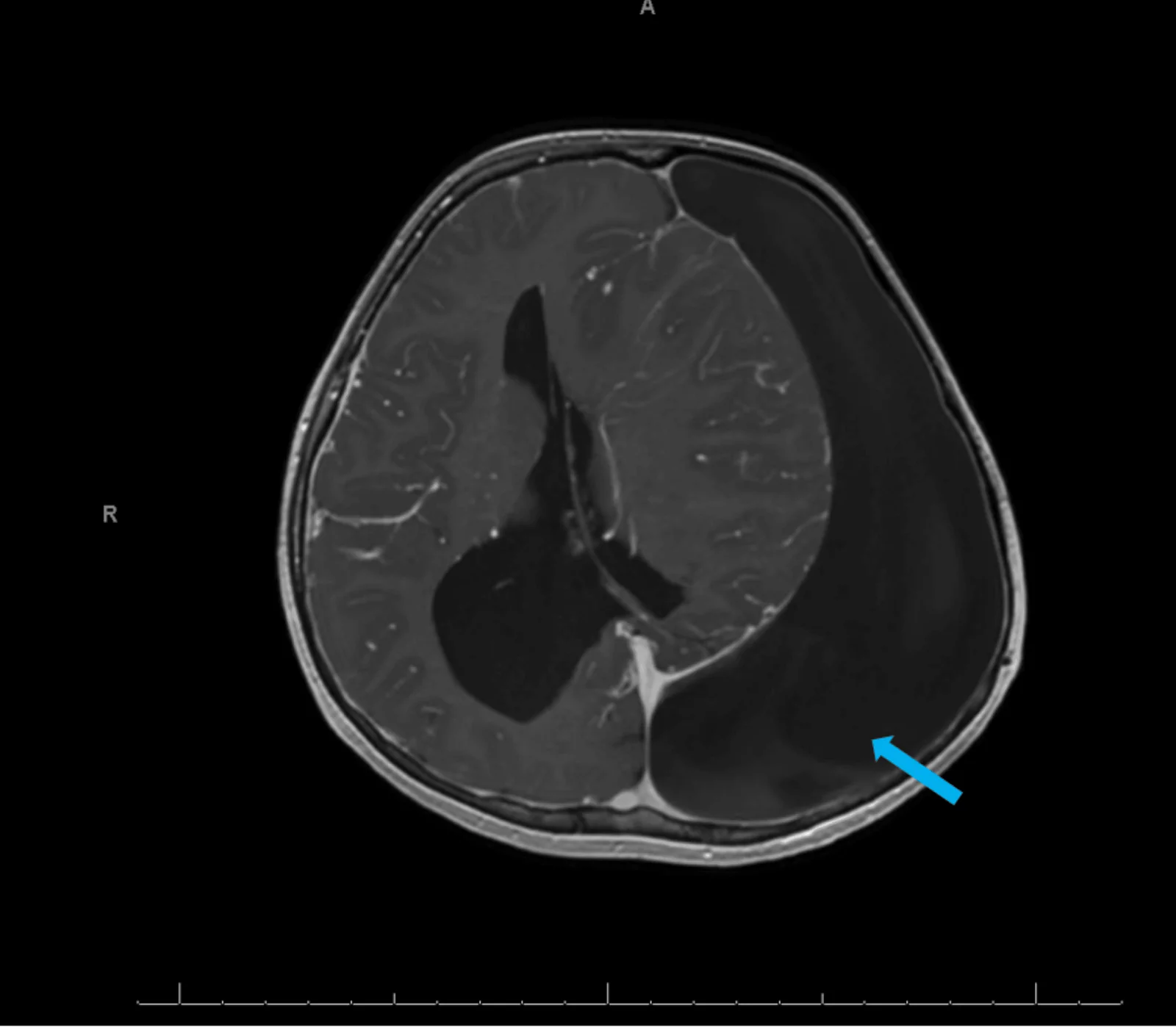
Most recent MRI (T1 post-contrast). Stable post-operative changes with resolution of the prior hematoma and a persistent but stable extra-axial fluid collection on the same side of resection (indicated by the blue arrow), with stable ventriculomegaly. No evidence of recurrent tumor noted.

The patient was started on single-agent NTRK-inhibitor larotrectinib 100 mg/m^2^ twice a day and continued this medication (with dose adjustments for weight gain) for 36 months. The medication was tolerated very well, with the only side effect being mild lymphopenia that required observation only; no dose reductions were necessary. The most recent follow-up (15 months after completion of larotrectinib and over five years after surgery) revealed stable post-operative changes status post resection of the tumor, with a similar-sized extra-axial fluid collection and enlarged ventricles (without hydrocephalus), and no residual tumor or new enhancing nodules (Figure 14). As the lesion is considered a high-grade glioma, spinal imaging is followed regularly for surveillance, which has remained negative. The patient has hyperopia and moderate astigmatism in both eyes, but healthy optic nerves and no visual field defects as of the most recent exam. The patient continues with regular physical therapy and equine therapy and has made remarkable improvements in all developmental domains.

## Discussion

Infant-type hemispheric glioma is a novel type of high-grade glioma that occurs in newborns and infants with a distinct molecular profile [3]. The 2021 WHO classification of CNS tumors has included a special category, pediatric-type diffuse gliomas, with two subcategories: low-grade gliomas and high-grade gliomas. Infant-type hemispheric glioma, classified as a high-grade glioma, is not currently numerically graded by WHO and is considered WHO grade 3/4 clinically. The essential diagnostic criteria to be met for the diagnosis of these tumors include the presence of early childhood presentation, cerebral hemispheric location, histological features of a cellular astrocytoma and either the presence of a methylation profile aligned with infant-type hemispheric glioma and/or the presence of a typical receptor tyrosine kinase abnormality (fusion in NTRK family gene or in ALK, MET1 or ROS1) [4,5].

The median age of presentation is 2.8 months, with a range of 0-12 months. Thus, most cases are seen during infancy [3,4]. Clinically, presentation can be variable, ranging from objective signs, such as large head circumference, to non-specific symptoms, such as lethargy and agitation. These gliomas are typically large in size, located in the supratentorial compartment [3,4,6-11], and can show leptomeningeal involvement [4,8].

Grossly, they can show solid and cystic components along with secondary changes such as hemorrhage or necrosis [3,4,6-11]. On microscopy, these tumors are comprised of fascicles or sheets of astrocytic and spindle-shaped cells displaying pleomorphism and frequent mitosis, palisading necrosis, microvascular proliferation, along with a focal desmoplastic component [1,7,9,11,12]. Gemistocytic cells, ganglion cells, and ependymal differentiation have also been reported. Prior to the new classification, these tumors were often diagnosed as glioblastomas or other high-grade gliomas [3,6-8,12]. Other differentials include anaplastic gangliogliomas, desmoplastic infantile ganglioglioma/astrocytoma, or CNS primitive neuroectodermal tumors [8,11].

Immunohistochemically, the tumor cells show positivity for GFAP, but neuronal markers are not expressed [10-12]. Variable positivity is seen with p53, whereas the tumor cells are negative for chromogranin, synaptophysin, NeuN, ATRX, BRAF V600E mutation, and H3K27M mutation [1,7,9,11,12]. Immunohistochemical positivity for ALK can be seen in some tumors with underlying ALK fusions. Due to high expression of NTRK in normal brain, this immunostain is not useful for diagnosing tumors with NTRK alterations [3,4,11].

Infant-type hemispheric gliomas show a very peculiar molecular profile based on a few studies conducted. Ruscio et al. identified the NTRK alteration in three out of 11 children (27%) with high-grade gliomas [2]. Clarke et al., while studying a unique intrinsic set of 130 infant hemispheric gliomas, reported fusions in either ALK, NTRK1/2/3, ROS1 or MET and identified targetable mitogen-activated protein kinase (MAPK) alterations. They found a better prognosis in kinase-fusion-positive tumors and clinical response to targeted therapy [2,8]. Guerriero et al. performed genetic analysis on a large international cohort of 150 cases of infantile high-grade gliomas (HGG) and identified three main groups. Group 1 gliomas arose in the cerebral hemispheres, showed alterations in receptor tyrosine kinases ALK, NTRK, ROS1, and MET, and had an intermediate prognosis. Group 2 showed good prognosis and long-term survival, and group 3 showed poor prognosis. Both groups had rat sarcoma (RAS)/MAPK pathway alterations and arose in the midline or in the cerebral hemispheres [2,3].

Tumors with NTRK gene fusions have gained significant interest due to their targetable nature and the recent availability of NTRK inhibitors. The most frequently reported NTRK fusion is ETS variant transcription factor 6 (ETV6)-NTRK3, in addition to other less frequent ones like echinoderm microtubule-associated protein-like 4 (EML)-NTRK3 and TPM3-NTRK1 fusions. All NTRK fusions are typically associated with the C-terminal kinase domain of NTRK 1/2/3 and the N-terminal domain of the partner gene, leading to the chimeric protein product activation [2,3,13].

Larotrectinib is a highly selective small-molecule inhibitor with activity against all tropomyosin receptor kinase (TRK) proteins in pre-clinical and subsequent clinical studies. It is well-tolerated in both pediatric and adult patients [14], with a safe pediatric dose having been established [15]. It was found to penetrate the blood-brain barrier and has shown promising results in TRK-driven HGG [8,12]. Additionally, it is available in a liquid formulation, which allows for use in very young patients.

Based on clinical and morphological findings, the differential diagnosis includes atypical teratoid rhabdoid tumor and desmoplastic infantile astrocytoma/ganglioglioma. All these tumors show the presence of undifferentiated cells [4,5,16]. Their similarities and differences with infant-type hemispheric glioma have been enumerated in Table 1. Other differentials include mesenchymal tumors and metastasis, which were ruled out in this case based on clinical presentation and immunohistochemical staining results.

**Table 1:** Differential diagnosis of infant-type hemispheric glioma and their similarities and differences. GFAP: glial fibrillary acidic protein; IHC: immunohistochemistry; MAP2: microtubule-associated protein 2; SMARCB1: SWI/SNF-related, matrix-associated, actin-dependent regulator of chromatin, subfamily B, member 1; EMA: epithelial membrane antigen.

	Atypical teratoid/rhabdoid tumor (ATRT)	Desmoplastic infantile astrocytoma/ganglioglioma (DIA) WHO 2016	Infant-type hemispheric glioma WHO 2021
Clinical (age)	<3 years	Infancy	Infancy, antenatally
Morphology	Rhabdoid cells, pale cells, undifferentiated cells, mitosis	Desmoplastic and poorly differentiated component (necrosis, mitosis)	Astrocytic (fascicles/sheets, mild/moderate pleomorphism), palisading necrosis, mitotic activity, microvascular proliferation
IHC	Positive stains: EMA, GFAP, synaptophysin	Positive stains: GFAP, vimentin, MAP2; Negative stains: pancytokeratin or EMA	Positive stains: GFAP; Negative stains: neuronal markers
Molecular	INI-1/SMARCB1 deletion	BRAFV600E mutations	NTRK family, ALK, ROS, MET
WHO grade	WHO grade IV	WHO grade I	WHO grade III-IV
Management and prognosis	Surgical resection and upfront radiation	Surgical resection	Being studied

Despite similarities in morphological features, CNS gliomas display a wide spectrum of molecular and epigenetic alterations. To guide correct classification and treatment of pediatric CNS tumors, histological features are not independently adequate [2,8]. Molecular profiling is essential to unveil the underlying biological alterations that characterize these tumors in order to utilize specific targeted therapies, facilitate more appropriate surveillance recommendations, and anticipate the likelihood of recurrence and/or resistance to therapies [2,17,18].

## Conclusions

Infant-type hemispheric glioma is a novel type of high-grade glioma recently defined by the updated 2021 WHO classification of central nervous system tumors under a special category, pediatric-type diffuse glioma. Fusions in either ALK, NTRK 1/2/3, ROS1 or MET and identified targetable MAPK alterations have been reported in these tumors. Larotrectinib, a highly selective small-molecule inhibitor with activity against all TRK proteins in pre-clinical and subsequent clinical studies, has shown promising results in TRK-driven HGG. Understanding the molecular profiles of these tumors could help utilize specific targeted therapies and facilitate appropriate management decisions.

## References

[1] Simone V, Rizzo D, Cocciolo A, Caroleo AM, Carai A, Mastronuzzi A, Tornesello A (2021). Infantile brain tumors: a review of literature and future perspectives. Diagnostics (Basel).

[2] Di Ruscio V, Carai A, Del Baldo G (2022). Molecular landscape in infant high-grade gliomas: a single center experience. Diagnostics (Basel).

[3] Guerreiro Stucklin AS, Ryall S, Fukuoka K (2019). Alterations in ALK/ROS1/NTRK/MET drive a group of infantile hemispheric gliomas. Nat Commun.

[4] WHO Classification of Tumours Editorial Board (2021). Central Nervous System Tumours. WHO Classification of Tumours, 5th ed. https://publications.iarc.who.int/Book-And-Report-Series/Who-Classification-Of-Tumours/Central-Nervous-System-Tumours-2021.

[5] Louis DN, Perry A, Wesseling P (2021). The 2021 WHO classification of tumors of the central nervous system: a summary. Neuro-Oncology.

[6] Aghajan Y, Levy ML, Malicki DM, Crawford JR (2016). Novel PPP1CB-ALK fusion protein in a high-grade glioma of infancy. BMJ Case Rep.

[7] Coccé MC, Mardin BR, Bens S (2016). Identification of ZCCHC8 as fusion partner of ROS1 in a case of congenital glioblastoma multiforme with a t(6;12)(q21;q24.3). Genes Chromosomes Cancer.

[8] Clarke M, Mackay A, Ismer B (2020). Infant high-grade gliomas comprise multiple subgroups characterized by novel targetable gene fusions and favorable outcomes. Cancer Discov.

[9] Ng A, Levy ML, Malicki DM, Crawford JR (2019). Unusual high-grade and low-grade glioma in an infant with PPP1CB-ALK gene fusion. BMJ Case Rep.

[10] Valera ET, Neder L, Queiroz RG (2020). Perinatal complex low- and high-grade glial tumor harboring a novel GIGYF2-ALK fusion. Pediatr Blood Cancer.

[11] Olsen TK, Panagopoulos I, Meling TR (2015). Fusion genes with ALK as recurrent partner in ependymoma-like gliomas: a new brain tumor entity?. Neuro-oncology.

[12] Ziegler DS, Wong M, Mayoh C (2018). Brief report: Potent clinical and radiological response to larotrectinib in TRK fusion-driven high-grade glioma. Br J Cancer.

[13] Harel L, Costa B, Fainzilber M (2010). On the death Trk. Dev Neurobiol.

[14] Ceglie G, Vinci M, Carai A (2020). Infantile/congenital high-grade gliomas: molecular features and therapeutic perspectives. Diagnostics (Basel).

[15] Hong DS, DuBois SG, Kummar S (2020). Larotrectinib in patients with TRK fusion-positive solid tumours: a pooled analysis of three phase 1/2 clinical trials. Lancet Oncol.

[16] Louis DN, Ohgaki H, Wiestler OD (2016). WHO Classification of Tumours of the Central Nervous System. https://publications.iarc.who.int/Book-And-Report-Series/Who-Classification-Of-Tumours/.

[17] Gusyatiner O, Hegi ME (2018). Glioma epigenetics: from subclassification to novel treatment options. Semin Cancer Biol.

[18] Dabrowski MJ, Wojtas B (2019). Global DNA methylation patterns in human gliomas and their interplay with other epigenetic modifications. Int J Mol Sci.

